# 9-(3-Fluoro­phen­oxy­carbon­yl)-10-methyl­acridinium trifluoro­methane­sulfonate monohydrate

**DOI:** 10.1107/S1600536812003054

**Published:** 2012-02-04

**Authors:** Damian Trzybiński, Agnieszka Ożóg, Karol Krzymiński, Jerzy Błażejowski

**Affiliations:** a Faculty of Chemistry, University of Gdańsk, J. Sobieskiego 18, 80-952 Gdańsk, Poland

## Abstract

In the crystal structure of the title mol­ecular salt, C_21_H_15_FNO_2_^+^·CF_3_SO_3_^−^·H_2_O, the cations form inversion dimers through π–π inter­actions between the acridine ring systems. These dimers are linked *via* C—H⋯O and C—F⋯π inter­actions to adjacent anions, and by C—H⋯π and C—F⋯π inter­actions to neighbouring cations. The water mol­ecule links two sites of the cation by C—H⋯O inter­actions and two adjacent anions by O—H⋯O hydrogen bonds. The mean planes of the acridine and benzene ring systems are oriented at a dihedral angle of 15.1 (1)°. The carboxyl group is twisted at an angle of 84.5 (1)° relative to the acridine skeleton. The mean planes of the acridine ring systems are parallel in the crystal.

## Related literature

For general background to the chemiluminogenic features of 9-phen­oxy­carbonyl-10-methyl­acridinium trifluoro­methane­sulfonates, see: King *et al.* (2007 ▶); Krzymiński *et al.* (2011 ▶); Roda *et al.* (2003 ▶). For related structures, see: Trzybiński *et al.* (2010 ▶). For inter­molecular inter­actions, see: Aakeröy *et al.* (1992 ▶); Dorn *et al.* (2005 ▶); Hunter *et al.* (2001 ▶); Novoa *et al.* (2006 ▶); Takahashi *et al.* (2001 ▶). For the synthesis, see: Sato (1996 ▶); Trzybiński *et al.* (2010 ▶).
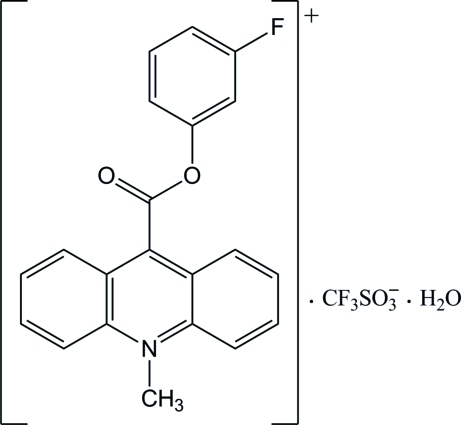


## Experimental

### 

#### Crystal data

C_21_H_15_FNO_2_^+^·CF_3_O_3_S^−^·H_2_O
*M* *_r_* = 499.44
Triclinic, 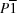

*a* = 9.5144 (10) Å
*b* = 11.5654 (11) Å
*c* = 11.9680 (12) Å
α = 109.975 (9)°
β = 97.838 (8)°
γ = 113.197 (9)°
*V* = 1080.3 (2) Å^3^
*Z* = 2
Mo *K*α radiation
μ = 0.23 mm^−1^
*T* = 295 K
0.40 × 0.15 × 0.10 mm

#### Data collection

Oxford Gemini R Ultra Ruby CCD diffractometer
9148 measured reflections
3769 independent reflections
1647 reflections with *I* > 2σ(*I*)
*R*_int_ = 0.050

#### Refinement

*R*[*F* ^2^ > 2σ(*F* ^2^)] = 0.051
*wR*(*F* ^2^) = 0.129
*S* = 0.81
3769 reflections
314 parameters
3 restraints
H atoms treated by a mixture of independent and constrained refinement
Δρ_max_ = 0.27 e Å^−3^
Δρ_min_ = −0.28 e Å^−3^

### 

Data collection: *CrysAlis CCD* (Oxford Diffraction, 2008 ▶); cell refinement: *CrysAlis RED* (Oxford Diffraction, 2008 ▶); data reduction: *CrysAlis RED*; program(s) used to solve structure: *SHELXS97* (Sheldrick, 2008 ▶); program(s) used to refine structure: *SHELXL97* (Sheldrick, 2008 ▶); molecular graphics: *ORTEP-3* (Farrugia, 1997 ▶); software used to prepare material for publication: *SHELXL97* and *PLATON* (Spek, 2009 ▶).

## Supplementary Material

Crystal structure: contains datablock(s) global, I. DOI: 10.1107/S1600536812003054/xu5452sup1.cif

Structure factors: contains datablock(s) I. DOI: 10.1107/S1600536812003054/xu5452Isup2.hkl

Supplementary material file. DOI: 10.1107/S1600536812003054/xu5452Isup3.cml

Additional supplementary materials:  crystallographic information; 3D view; checkCIF report

## Figures and Tables

**Table 1:** Hydrogen-bond geometry (Å, °) *Cg*4 is the centroid of the C18–C23 ring.

*D*—H⋯*A*	*D*—H	H⋯*A*	*D*⋯*A*	*D*—H⋯*A*
O1*W*—H1*W*⋯O29^i^	0.85 (3)	2.24 (3)	3.071 (5)	172 (4)
O1*W*—H2*W*⋯O28	0.89 (3)	1.99 (3)	2.873 (5)	176 (8)
C1—H1⋯O1*W*	0.93	2.51	3.365 (7)	152
C3—H3⋯O29^ii^	0.93	2.60	3.298 (5)	133
C19—H19⋯O1*W*	0.93	2.60	3.415 (7)	145
C25—H25*A*⋯O27^iii^	0.96	2.53	3.424 (5)	155
C25—H25*C*⋯*Cg*4^ii^	0.96	2.64	3.527 (4)	154

Symmetry codes: (i) 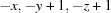; (ii) 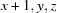; (iii) 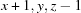.

**Table 2:** C—F⋯π inter­actions (Å,°) *Cg*1, *Cg*2 and *Cg*3 are the centroids of the C9/N10/C11–C14, C1–C4/C11/C12 and C5–C8/C13/C14 rings, respectively.

*X*	*I*	*J*	*I*⋯*J*	*X*⋯*J*	*X*—*I*⋯*J*
C20	F24	*Cg*2^iv^	3.743 (3)	4.139 (5)	97.6 (2)
C20	F24	*Cg*2^v^	3.854 (4)	4.188 (5)	94.9 (3)
C30	F31	*Cg*1^v^	3.665 (4)	4.519 (6)	123.6 (3)
C30	F31	*Cg*3^v^	3.910 (4)	4.049 (6)	86.7 (3)
C30	F33	*Cg*3^v^	3.654 (4)	4.049 (6)	97.7 (3)

Symmetry codes: (iv) *x* − 1, *y*, *z*; (v) −*x* + 1, −*y* + 2, −*z* + 1.

**Table 3:** π–π inter­actions (Å,°) *Cg*1, *Cg*2 and *Cg*3 are as defined in Table 2. *CgI*⋯*CgJ* is the distance between ring centroids. The dihedral angle is that between the planes of the rings *I* and *J. CgI*_Perp_ is the perpendicular distance of *CgI* from ring *J. CgI*_Offset_ is the distance between *CgI* and the perpendicular projection of *CgJ* on ring *I*.

*I*	*J*	*CgI*⋯*CgJ*	Dihedral angle	*CgI*_Perp_	*CgI*_Offset_
1	1^vi^	3.990 (2)		3.591 (2)	1.739 (2)
1	3^vi^	3.645 (2)	2.08 (17)	3.557 (2)	0.796 (2)
2	3^vi^	3.907 (2)	3.85 (19)	3.431 (2)	1.863 (2)
3	1^vi^	3.645 (2)	2.08 (17)	3.546 (2)	0.844 (2)
3	2^vi^	3.907 (2)	3.85 (19)	3.548 (2)	1.629 (2)

Symmetry code: (vi) −*x* + 1, −*y* + 1, −*z*.

**Fig. 1.**
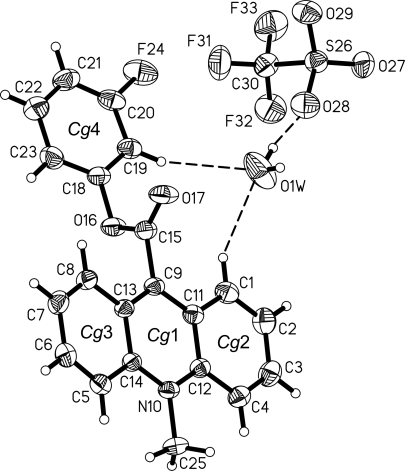
The molecular structure of the title compound showing the atom labeling scheme. Displacement ellipsoids are drawn at the 25% probability level and H atoms are shown as small spheres of arbitrary radius. Cg1, Cg2, Cg3 and Cg4 denote the ring centroids. The O—H···O and C—H···O hydrogen bonds are represented by dashed lines.

**Fig. 2.**
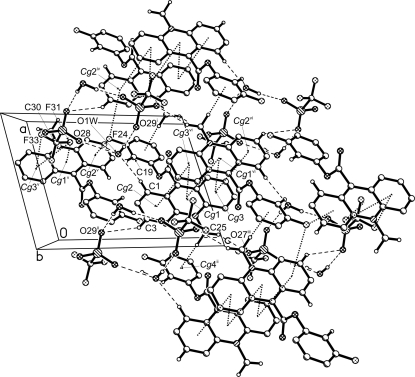
The arrangement of the ions and H2O molecules in the crystal structure, viewed along b-direction. The O—H···O and C—H···O interactions are represented by dashed lines, the C—H···π, C—F···π and π—π contacts by dotted lines. H atoms not involved in interactions have been omitted. [Symmetry codes: (i) -x, -y + 1, -z + 1; (ii) x + 1, y, z; (iii) x + 1, y, z - 1; (iv) x - 1, y, z; (v) -x + 1, -y + 2, -z + 1; (vi) -x + 1, -y + 1, -z.]

## supplementary crystallographic information

### Comment

9-Phenoxycarbonyl-10-methylacridinium cations react with oxidants (e.g. H_2_O_2_) in alkaline media, as a result of which electronically excited 10-methyl-9-acridinone molecules are generated (Krzymiński *et al.*, 2011). This phenomenon forms the basis for the use of these entities as chemiluminogenic indicators or fragments of chemiluminescent labels (Roda *et al.*, 2003; King *et al.*, 2007; Krzymiński *et al.*, 2011). It has been noted that the conversion efficiency of the above-mentioned cations to 10-methyl-9-acridinone molecules, and consequently the chemiluminescence quantum yield, crucial in analytical applications, depends on the structure of the phenoxycarbonyl fragment (Krzymiński *et al.*, 2011). For these reasons we have been synthesizing 9-phenoxycarbonyl-10-methylacridinium trifluoromethanesulfonates variously substituted in the phenyl fragment in order to select derivatives optimal for analytical applications. Here we present the structure of one of the compounds of this series.

In the cation of the title compound (Fig. 1), the bond lengths and angles characterizing the geometry of the acridinium and phenyl moieties are typical of 9-phenoxycarbonyl-10-methylacridinium derivatives (Trzybiński *et al.*, 2010). With respective average deviations from planarity of 0.0397 (3) Å and 0.0066 (3) Å, the acridine and benzene ring systems are oriented at a dihedral angle of 15.1 (1)° [in 9-(4-fluorophenoxycarbonyl)-10-methylacridinium trifluoromethanesulfonate this angle is equal to 74.1 (1)° (Trzybiński *et al.*, 2010)]. The carboxyl group is twisted at an angle of 84.5 (1)° relative to the acridine skeleton [in 9-(4-fluorophenoxycarbonyl)-10-methylacridinium trifluoromethanesulfonate this angle is 4.4 (1)° (Trzybiński *et al.*, 2010)]. The mean planes of the adjacent acridine moieties are parallel [remain at an angle of 0.0 (1)°)] in the lattice.

In the crystal structure, the inversely oriented cations form dimers through π–π contacts involving all three rings of the acridine aromatic system (Table 3, Fig. 2). These dimers are linked by C—H···O (Table 1, Fig. 2) and C—F···π (Table 2, Fig. 2) interactions to adjacent anions and by C—H···π (Table 1, Fig. 2) and C—F···π (Table 2, Fig. 2) interactions to neighbouring cations. Each cation is involved in two C—H···O interactions with a water molecule, which in turn is engaged in O—H···O hydrogen bonds involving O atoms of two adjacent anions (Table 1, Figs. 1 and 2). The O—H···O (Aakeröy *et al.*, 1992) and C—H···O (Novoa *et al.*, 2006) interactions are of the hydrogen bond type. The C—H···π interactions should be of an attractive nature (Takahashi *et al.*, 2001), like the C—F···π (Dorn *et al.*, 2005) and the π–π (Hunter *et al.*, 2001) interactions. The crystal structure is stabilized by a network of these short-range specific interactions and by long-range electrostatic interactions between ions.

### Experimental

3-Fluorophenylacridine-9-carboxylate was obtained by esterification of 9-(chlorocarbonyl)acridine (synthesized in the reaction of acridine-9-carboxylic acid with a tenfold excess of thionyl chloride) with 3-fluorophenol in anhydrous dichloromethane in the presence of N,N-diethylethanamine and a catalytic amount of N,N-dimethyl-4-pyridinamine (room temperature, 15 h) (Sato, 1996). The product was purified chromatographically (SiO_2_, cyclohexane/ethyl acetate, 1/1 v/v) and subsequently quaternarized with a fivefold molar excess of methyl trifluoromethanesulfonate dissolved in anhydrous dichloromethane. The crude 3-(fluorophenoxycarbonyl)-10-methylacridinium trifluoromethanesulfonate was dissolved in a small amount of ethanol, filtered and precipitated with a 20 v/v excess of diethyl ether (Trzybiński *et al.*, 2010). Light-yellow crystals suitable for X-ray investigations were grown from methanol/water solution (1/1 v/v) (m.p. 497–498 K).

### Refinement

The H atoms of the water molecule were located on a Fourier difference map, restrained by DFIX command 0.85 for O—H distances and by DFIX 1.39 for H···H distance, and refined as riding with *U*_iso_(H) = 1.5*U*_eq_(O). Other H atoms were positioned geometrically, with C—H = 0.93 Å and 0.96 Å for the aromatic and methyl H atoms, respectively, and constrained to ride on their parent atoms with *U*_iso_(H) = *xU*_eq_(C), where *x* = 1.2 for the aromatic H atoms and *x* = 1.5 for the methyl H atoms.

### Crystal data

**Table:**

C_21_H_15_FNO_2_^+^·CF_3_O_3_S^−^·H_2_O	*Z* = 2
*M**_r_* = 499.44	*F*(000) = 512
Triclinic, *P*1	*D*_x_ = 1.535 Mg m^−^^3^
Hall symbol: -P 1	Mo *K*α radiation, λ = 0.71073 Å
*a* = 9.5144 (10) Å	Cell parameters from 2551 reflections
*b* = 11.5654 (11) Å	θ = 3.1–29.0°
*c* = 11.9680 (12) Å	µ = 0.23 mm^−^^1^
α = 109.975 (9)°	*T* = 295 K
β = 97.838 (8)°	Prism, light yellow
γ = 113.197 (9)°	0.40 × 0.15 × 0.10 mm
*V* = 1080.3 (2) Å^3^	

### Data collection

**Table:**

Oxford Gemini R Ultra Ruby CCD diffractometer	1647 reflections with *I* > 2σ(*I*)
Radiation source: Enhanced (Mo) X-ray Source	*R*_int_ = 0.050
Graphite monochromator	θ_max_ = 25.1°, θ_min_ = 3.1°
Detector resolution: 10.4002 pixels mm^-1^	*h* = −11→11
ω scans	*k* = −13→13
9148 measured reflections	*l* = −13→14
3769 independent reflections	

### Refinement

**Table:**

Refinement on *F*^2^	Primary atom site location: structure-invariant direct methods
Least-squares matrix: full	Secondary atom site location: difference Fourier map
*R*[*F*^2^ > 2σ(*F*^2^)] = 0.051	Hydrogen site location: inferred from neighbouring sites
*wR*(*F*^2^) = 0.129	H atoms treated by a mixture of independent and constrained refinement
*S* = 0.81	*w* = 1/[σ^2^(*F*_o_^2^) + (0.0706*P*)^2^] where *P* = (*F*_o_^2^ + 2*F*_c_^2^)/3
3769 reflections	(Δ/σ)_max_ = 0.003
314 parameters	Δρ_max_ = 0.27 e Å^−^^3^
3 restraints	Δρ_min_ = −0.28 e Å^−^^3^

### Special details

**Table:**

Geometry. All esds (except the esd in the dihedral angle between two l.s. planes) are estimated using the full covariance matrix. The cell esds are taken into account individually in the estimation of esds in distances, angles and torsion angles; correlations between esds in cell parameters are only used when they are defined by crystal symmetry. An approximate (isotropic) treatment of cell esds is used for estimating esds involving l.s. planes.

### Fractional atomic coordinates and isotropic or equivalent isotropic displacement parameters (Å^2^)

**Table:**

	*x*	*y*	*z*	*U*_iso_*/*U*_eq_	
C1	0.6064 (4)	0.6768 (4)	0.3644 (3)	0.0694 (10)	
H1	0.5226	0.6930	0.3842	0.083*	
O1W	0.2866 (5)	0.6414 (4)	0.4672 (4)	0.1503 (14)	
H1W	0.229 (7)	0.553 (3)	0.420 (5)	0.225*	
H2W	0.258 (8)	0.655 (6)	0.535 (4)	0.225*	
C2	0.6940 (5)	0.6521 (4)	0.4419 (3)	0.0774 (11)	
H2	0.6694	0.6494	0.5139	0.093*	
C3	0.8221 (5)	0.6303 (4)	0.4147 (4)	0.0780 (11)	
H3	0.8836	0.6155	0.4703	0.094*	
C4	0.8587 (4)	0.6302 (4)	0.3100 (3)	0.0669 (9)	
H4	0.9441	0.6147	0.2938	0.080*	
C5	0.7416 (4)	0.6647 (3)	−0.0808 (3)	0.0610 (9)	
H5	0.8279	0.6514	−0.0982	0.073*	
C6	0.6469 (5)	0.6792 (4)	−0.1633 (3)	0.0701 (10)	
H6	0.6688	0.6748	−0.2376	0.084*	
C7	0.5172 (4)	0.7005 (4)	−0.1417 (3)	0.0712 (10)	
H7	0.4535	0.7095	−0.2011	0.085*	
C8	0.4848 (4)	0.7081 (3)	−0.0346 (3)	0.0619 (9)	
H8	0.3984	0.7228	−0.0200	0.074*	
C9	0.5504 (4)	0.7021 (3)	0.1689 (3)	0.0519 (8)	
N10	0.8010 (3)	0.6524 (3)	0.1173 (2)	0.0524 (7)	
C11	0.6393 (4)	0.6788 (3)	0.2530 (3)	0.0539 (8)	
C12	0.7684 (4)	0.6534 (3)	0.2251 (3)	0.0521 (8)	
C13	0.5797 (3)	0.6941 (3)	0.0564 (3)	0.0502 (8)	
C14	0.7103 (4)	0.6698 (3)	0.0315 (3)	0.0501 (8)	
C15	0.4158 (5)	0.7316 (4)	0.1978 (3)	0.0609 (9)	
O16	0.4730 (3)	0.8678 (2)	0.2685 (2)	0.0703 (7)	
O17	0.2782 (3)	0.6459 (3)	0.1585 (3)	0.0872 (8)	
C18	0.3576 (4)	0.9124 (3)	0.2952 (3)	0.0613 (9)	
C19	0.3045 (4)	0.9060 (4)	0.3932 (3)	0.0672 (9)	
H19	0.3389	0.8693	0.4426	0.081*	
C20	0.1975 (4)	0.9564 (4)	0.4164 (4)	0.0749 (10)	
C21	0.1428 (5)	1.0080 (4)	0.3448 (4)	0.0842 (12)	
H21	0.0687	1.0395	0.3624	0.101*	
C22	0.1981 (5)	1.0130 (4)	0.2468 (4)	0.0915 (13)	
H22	0.1613	1.0478	0.1966	0.110*	
C23	0.3089 (5)	0.9664 (4)	0.2211 (4)	0.0816 (11)	
H23	0.3492	0.9716	0.1554	0.098*	
F24	0.1452 (3)	0.9541 (3)	0.5144 (2)	0.1166 (9)	
C25	0.9405 (4)	0.6317 (4)	0.0943 (3)	0.0695 (10)	
H25A	0.9560	0.6413	0.0197	0.104*	
H25B	0.9204	0.5401	0.0843	0.104*	
H25C	1.0355	0.7005	0.1642	0.104*	
S26	0.08915 (12)	0.70895 (10)	0.76540 (9)	0.0699 (3)	
O27	0.1099 (3)	0.6733 (3)	0.8669 (2)	0.0903 (8)	
O28	0.1777 (3)	0.6826 (3)	0.6828 (2)	0.1036 (9)	
O29	−0.0729 (3)	0.6712 (3)	0.7054 (2)	0.0931 (8)	
C30	0.1857 (6)	0.8973 (5)	0.8431 (4)	0.0860 (12)	
F31	0.1788 (4)	0.9507 (3)	0.7638 (3)	0.1385 (10)	
F32	0.3375 (3)	0.9479 (3)	0.9058 (3)	0.1292 (9)	
F33	0.1148 (4)	0.9392 (3)	0.9240 (3)	0.1307 (10)	

### Atomic displacement parameters (Å^2^)

**Table:**

	*U*^11^	*U*^22^	*U*^33^	*U*^12^	*U*^13^	*U*^23^
C1	0.066 (3)	0.071 (3)	0.065 (2)	0.030 (2)	0.023 (2)	0.025 (2)
O1W	0.182 (4)	0.113 (3)	0.147 (3)	0.046 (3)	0.105 (3)	0.056 (3)
C2	0.093 (3)	0.077 (3)	0.058 (2)	0.037 (3)	0.021 (2)	0.029 (2)
C3	0.084 (3)	0.074 (3)	0.070 (3)	0.039 (2)	0.009 (2)	0.030 (2)
C4	0.062 (2)	0.070 (3)	0.068 (2)	0.034 (2)	0.015 (2)	0.028 (2)
C5	0.056 (2)	0.059 (2)	0.063 (2)	0.023 (2)	0.021 (2)	0.0251 (19)
C6	0.075 (3)	0.069 (3)	0.066 (2)	0.029 (2)	0.024 (2)	0.034 (2)
C7	0.069 (3)	0.073 (3)	0.074 (3)	0.033 (2)	0.013 (2)	0.039 (2)
C8	0.054 (2)	0.063 (2)	0.073 (2)	0.030 (2)	0.018 (2)	0.031 (2)
C9	0.0383 (19)	0.041 (2)	0.063 (2)	0.0149 (17)	0.0100 (18)	0.0155 (17)
N10	0.0393 (16)	0.0494 (17)	0.0600 (17)	0.0198 (14)	0.0128 (14)	0.0170 (14)
C11	0.049 (2)	0.047 (2)	0.055 (2)	0.0186 (18)	0.0146 (18)	0.0156 (17)
C12	0.046 (2)	0.047 (2)	0.053 (2)	0.0194 (18)	0.0093 (17)	0.0160 (17)
C13	0.0399 (19)	0.046 (2)	0.058 (2)	0.0177 (17)	0.0096 (17)	0.0199 (17)
C14	0.0427 (19)	0.043 (2)	0.057 (2)	0.0176 (17)	0.0115 (17)	0.0187 (17)
C15	0.052 (2)	0.062 (3)	0.069 (2)	0.029 (2)	0.019 (2)	0.026 (2)
O16	0.0491 (15)	0.0527 (17)	0.0950 (18)	0.0241 (14)	0.0246 (14)	0.0159 (14)
O17	0.0458 (17)	0.0675 (18)	0.120 (2)	0.0229 (16)	0.0242 (16)	0.0149 (16)
C18	0.047 (2)	0.050 (2)	0.075 (2)	0.0228 (19)	0.019 (2)	0.0160 (19)
C19	0.053 (2)	0.062 (2)	0.070 (2)	0.023 (2)	0.017 (2)	0.017 (2)
C20	0.063 (2)	0.078 (3)	0.067 (3)	0.029 (2)	0.029 (2)	0.015 (2)
C21	0.076 (3)	0.075 (3)	0.101 (3)	0.047 (3)	0.030 (3)	0.024 (3)
C22	0.088 (3)	0.091 (3)	0.115 (4)	0.054 (3)	0.042 (3)	0.049 (3)
C23	0.084 (3)	0.079 (3)	0.099 (3)	0.043 (3)	0.050 (3)	0.044 (3)
F24	0.1057 (19)	0.148 (2)	0.0961 (18)	0.0634 (18)	0.0560 (16)	0.0400 (16)
C25	0.053 (2)	0.084 (3)	0.077 (2)	0.042 (2)	0.023 (2)	0.029 (2)
S26	0.0717 (7)	0.0774 (7)	0.0647 (6)	0.0376 (6)	0.0255 (6)	0.0312 (5)
O27	0.104 (2)	0.111 (2)	0.0891 (19)	0.0613 (19)	0.0385 (17)	0.0646 (18)
O28	0.124 (2)	0.117 (2)	0.099 (2)	0.073 (2)	0.067 (2)	0.0466 (18)
O29	0.0619 (18)	0.116 (2)	0.0755 (18)	0.0298 (17)	0.0063 (14)	0.0341 (17)
C30	0.084 (3)	0.096 (3)	0.093 (3)	0.050 (3)	0.028 (3)	0.048 (3)
F31	0.149 (3)	0.122 (2)	0.169 (3)	0.059 (2)	0.039 (2)	0.101 (2)
F32	0.0778 (19)	0.102 (2)	0.152 (2)	0.0157 (16)	−0.0044 (17)	0.0420 (18)
F33	0.152 (3)	0.104 (2)	0.129 (2)	0.077 (2)	0.047 (2)	0.0220 (17)

### Geometric parameters (Å, º)

**Table:**

C1—C2	1.340 (4)	C11—C12	1.427 (4)
C1—C11	1.416 (4)	C13—C14	1.425 (4)
C1—H1	0.9300	C15—O17	1.187 (4)
O1W—H1W	0.86 (2)	C15—O16	1.335 (4)
O1W—H2W	0.874 (19)	O16—C18	1.415 (3)
C2—C3	1.396 (5)	C18—C19	1.352 (4)
C2—H2	0.9300	C18—C23	1.373 (5)
C3—C4	1.345 (4)	C19—C20	1.375 (4)
C3—H3	0.9300	C19—H19	0.9300
C4—C12	1.404 (4)	C20—F24	1.339 (4)
C4—H4	0.9300	C20—C21	1.354 (5)
C5—C6	1.343 (4)	C21—C22	1.359 (5)
C5—C14	1.404 (4)	C21—H21	0.9300
C5—H5	0.9300	C22—C23	1.385 (5)
C6—C7	1.390 (4)	C22—H22	0.9300
C6—H6	0.9300	C23—H23	0.9300
C7—C8	1.342 (4)	C25—H25A	0.9600
C7—H7	0.9300	C25—H25B	0.9600
C8—C13	1.412 (4)	C25—H25C	0.9600
C8—H8	0.9300	S26—O28	1.415 (2)
C9—C13	1.391 (4)	S26—O27	1.423 (2)
C9—C11	1.391 (4)	S26—O29	1.427 (2)
C9—C15	1.504 (4)	S26—C30	1.806 (5)
N10—C12	1.365 (4)	C30—F31	1.304 (4)
N10—C14	1.369 (4)	C30—F32	1.315 (4)
N10—C25	1.484 (3)	C30—F33	1.320 (4)
			
C2—C1—C11	121.1 (3)	C5—C14—C13	118.5 (3)
C2—C1—H1	119.4	O17—C15—O16	125.6 (3)
C11—C1—H1	119.4	O17—C15—C9	124.2 (3)
H1W—O1W—H2W	105 (3)	O16—C15—C9	110.2 (3)
C1—C2—C3	120.1 (3)	C15—O16—C18	116.3 (3)
C1—C2—H2	120.0	C19—C18—C23	122.6 (3)
C3—C2—H2	120.0	C19—C18—O16	120.1 (3)
C4—C3—C2	121.6 (3)	C23—C18—O16	117.2 (3)
C4—C3—H3	119.2	C18—C19—C20	116.8 (3)
C2—C3—H3	119.2	C18—C19—H19	121.6
C3—C4—C12	120.3 (3)	C20—C19—H19	121.6
C3—C4—H4	119.8	F24—C20—C21	118.8 (3)
C12—C4—H4	119.8	F24—C20—C19	118.2 (4)
C6—C5—C14	119.9 (3)	C21—C20—C19	123.0 (4)
C6—C5—H5	120.0	C20—C21—C22	118.8 (3)
C14—C5—H5	120.0	C20—C21—H21	120.6
C5—C6—C7	122.4 (3)	C22—C21—H21	120.6
C5—C6—H6	118.8	C21—C22—C23	120.4 (4)
C7—C6—H6	118.8	C21—C22—H22	119.8
C8—C7—C6	119.4 (3)	C23—C22—H22	119.8
C8—C7—H7	120.3	C18—C23—C22	118.2 (4)
C6—C7—H7	120.3	C18—C23—H23	120.9
C7—C8—C13	121.2 (3)	C22—C23—H23	120.9
C7—C8—H8	119.4	N10—C25—H25A	109.5
C13—C8—H8	119.4	N10—C25—H25B	109.5
C13—C9—C11	121.5 (3)	H25A—C25—H25B	109.5
C13—C9—C15	119.1 (3)	N10—C25—H25C	109.5
C11—C9—C15	119.4 (3)	H25A—C25—H25C	109.5
C12—N10—C14	122.5 (2)	H25B—C25—H25C	109.5
C12—N10—C25	117.7 (2)	O28—S26—O27	115.73 (15)
C14—N10—C25	119.8 (3)	O28—S26—O29	114.66 (17)
C9—C11—C1	123.2 (3)	O27—S26—O29	115.01 (16)
C9—C11—C12	118.6 (3)	O28—S26—C30	102.91 (19)
C1—C11—C12	118.2 (3)	O27—S26—C30	102.55 (19)
N10—C12—C4	121.9 (3)	O29—S26—C30	103.36 (18)
N10—C12—C11	119.4 (3)	F31—C30—F32	109.0 (4)
C4—C12—C11	118.7 (3)	F31—C30—F33	107.7 (3)
C9—C13—C8	122.8 (3)	F32—C30—F33	107.5 (4)
C9—C13—C14	118.6 (3)	F31—C30—S26	111.8 (3)
C8—C13—C14	118.6 (3)	F32—C30—S26	110.4 (3)
N10—C14—C5	122.2 (3)	F33—C30—S26	110.3 (3)
N10—C14—C13	119.3 (3)		
			
C11—C1—C2—C3	1.2 (6)	C6—C5—C14—C13	−1.7 (5)
C1—C2—C3—C4	−1.6 (6)	C9—C13—C14—N10	1.2 (4)
C2—C3—C4—C12	0.5 (6)	C8—C13—C14—N10	−178.4 (3)
C14—C5—C6—C7	0.6 (5)	C9—C13—C14—C5	−178.6 (3)
C5—C6—C7—C8	0.4 (5)	C8—C13—C14—C5	1.8 (4)
C6—C7—C8—C13	−0.3 (5)	C13—C9—C15—O17	81.3 (5)
C13—C9—C11—C1	−175.7 (3)	C11—C9—C15—O17	−96.5 (4)
C15—C9—C11—C1	2.1 (5)	C13—C9—C15—O16	−95.9 (3)
C13—C9—C11—C12	3.3 (5)	C11—C9—C15—O16	86.3 (4)
C15—C9—C11—C12	−178.9 (3)	O17—C15—O16—C18	−1.8 (5)
C2—C1—C11—C9	179.1 (3)	C9—C15—O16—C18	175.4 (3)
C2—C1—C11—C12	0.1 (5)	C15—O16—C18—C19	85.5 (4)
C14—N10—C12—C4	177.5 (3)	C15—O16—C18—C23	−97.1 (4)
C25—N10—C12—C4	−2.5 (4)	C23—C18—C19—C20	0.1 (5)
C14—N10—C12—C11	−2.5 (4)	O16—C18—C19—C20	177.4 (3)
C25—N10—C12—C11	177.4 (3)	C18—C19—C20—F24	−178.7 (3)
C3—C4—C12—N10	−179.2 (3)	C18—C19—C20—C21	1.3 (6)
C3—C4—C12—C11	0.8 (5)	F24—C20—C21—C22	178.7 (3)
C9—C11—C12—N10	−0.1 (4)	C19—C20—C21—C22	−1.3 (6)
C1—C11—C12—N10	178.9 (3)	C20—C21—C22—C23	−0.2 (6)
C9—C11—C12—C4	179.8 (3)	C19—C18—C23—C22	−1.5 (6)
C1—C11—C12—C4	−1.1 (4)	O16—C18—C23—C22	−178.9 (3)
C11—C9—C13—C8	175.7 (3)	C21—C22—C23—C18	1.6 (6)
C15—C9—C13—C8	−2.0 (5)	O28—S26—C30—F31	59.7 (3)
C11—C9—C13—C14	−3.9 (4)	O27—S26—C30—F31	−179.8 (3)
C15—C9—C13—C14	178.4 (3)	O29—S26—C30—F31	−60.0 (3)
C7—C8—C13—C9	179.6 (3)	O28—S26—C30—F32	−61.8 (3)
C7—C8—C13—C14	−0.8 (5)	O27—S26—C30—F32	58.7 (3)
C12—N10—C14—C5	−178.2 (3)	O29—S26—C30—F32	178.6 (3)
C25—N10—C14—C5	1.9 (4)	O28—S26—C30—F33	179.5 (3)
C12—N10—C14—C13	2.0 (4)	O27—S26—C30—F33	−60.0 (3)
C25—N10—C14—C13	−178.0 (3)	O29—S26—C30—F33	59.8 (3)
C6—C5—C14—N10	178.5 (3)		

### Hydrogen-bond geometry (Å, º)

*Cg*4 is the centroid of the C18–C23 ring.

**Table:**

*D*—H···*A*	*D*—H	H···*A*	*D*···*A*	*D*—H···*A*
O1*W*—H1*W*···O29^i^	0.85 (3)	2.24 (3)	3.071 (5)	172 (4)
O1*W*—H2*W*···O28	0.89 (3)	1.99 (3)	2.873 (5)	176 (8)
C1—H1···O1*W*	0.93	2.51	3.365 (7)	152
C3—H3···O29^ii^	0.93	2.60	3.298 (5)	133
C19—H19···O1*W*	0.93	2.60	3.415 (7)	145
C25—H25*A*···O27^iii^	0.96	2.53	3.424 (5)	155
C25—H25*C*···*Cg*4^ii^	0.96	2.64	3.527 (4)	154

Symmetry codes: (i) −*x*, −*y*+1, −*z*+1; (ii) *x*+1, *y*, *z*; (iii) *x*+1, *y*, *z*−1.

### C—F···π interactions (Å,°)

**Table:**

*X*	*I*	*J*	*I*···*J*	*X*···*J*	*X*—*I*···*J*
C20	F24	*Cg*2^iv^	3.743 (3)	4.139 (5)	97.6 (2)
C20	F24	*Cg*2^v^	3.854 (4)	4.188 (5)	94.9 (3)
C30	F31	*Cg*1^v^	3.665 (4)	4.519 (6)	123.6 (3)
C30	F31	*Cg*3^v^	3.910 (4)	4.049 (6)	86.7 (3)
C30	F33	*Cg*3^v^	3.654 (4)	4.049 (6)	97.7 (3)

Symmetry codes: (iv) *x* - 1, *y*, *z*; (v) -*x* + 1, -*y* + 2, -*z* + 1. *Cg*1, *Cg*2 and *Cg*3 are the centroids of the C9/N10/C11–C14, C1–C4/C11/C12 and C5–C8/C13/C14 rings, respectively.

### π–π interactions (Å,°)

**Table:**

*I*	*J*	*CgI*···*CgJ*	Dihedral angle	*CgI*_Perp_	Cg*I*_Offset_
1	1^vi^	3.990 (2)	0	3.591 (2)	1.739 (2)
1	3^vi^	3.645 (2)	2.08 (17)	3.557 (2)	0.796 (2)
2	3^vi^	3.907 (2)	3.85 (19)	3.431 (2)	1.863 (2)
3	1^vi^	3.645 (2)	2.08 (17)	3.546 (2)	0.844 (2)
3	2^vi^	3.907 (2)	3.85 (19)	3.548 (2)	1.629 (2)

Symmetry codes: (vi) -*x* + 1, -*y* + 1, -*z*. Notes: *Cg*1, *Cg*2 and *Cg*3 are the centroids of the C9/N10/C11–C14, C1–C4/C11/C12 and C5–C8/C13/C14 rings, respectively. Cg*I*···Cg*J* is the distance between ring centroids. The dihedral angle is that between the planes of the rings *I* and *J*. *CgI*_Perp_ is the perpendicular distance of *CgI* from ring *J*. Cg*I*_Offset_ is the distance between *CgI* and the perpendicular projection of *CgJ* on ring *I*.
